# The Potential Future of Insects in the European Food System: A Systematic Review Based on the Consumer Point of View

**DOI:** 10.3390/foods12030646

**Published:** 2023-02-02

**Authors:** Giorgio Mina, Giovanni Peira, Alessandro Bonadonna

**Affiliations:** Department of Management, University of Turin, Corso Unione Sovietica 218 bis, 10134 Turin, Italy

**Keywords:** insect food, edible insect, entomophagy, consumer perception, sustainable protein, meat substitutes

## Abstract

Edible insects recently gained attention as a potential contributor to the future sustainability of the food system. Insect farming has indeed shown to have environmental and nutritional benefits, but edible insects are still an unusual foodstuff in Europe. The purpose of this article is to analyze the barriers and drivers of insect consumption in Europe and to identify the most promising strategies to convince consumers to include insect-based products in their diets. To answer these research questions, a systematic review of the literature on the consumer’s point of view about insects as food was performed. The results show that the main barrier to the development of this market is related to the psychological rejection of consumers induced by disgust toward entomophagy. To break down these barriers, it is essential to increase the general knowledge about the environmental and nutritional benefits of entomophagy. Furthermore, the limited size of the edible insect market appears to be a structural barrier. Expanding the reach of the market and consumer familiarity with edible insects will increase their acceptability. Finally, some product-related strategies are also highlighted. Furthermore, this article brings new knowledge about the effectiveness of the environmental motive in convincing consumers to try edible insects.

## 1. Introduction

The global demand for food products continues to grow, reflecting dietary changes, driven by population growth, a rise in income and increased urbanization [1]. Furthermore, the Food and Agriculture Organization (FAO) estimates that global food production should increase by 70 percent. Consequently, it will increase the exploitation of natural resources, such as land, water, and energy. This situation could produce a worldwide lack of protein. To prevent a global supply-demand gap the production of protein will need to increase by around 40% in the next 30 years [2]. The great challenge of this century lies in finding solutions to feed a growing population and, at the same time, reduce the impact of the food system on the environment [3]. These reasons could be the key drivers behind the development of the edible insect sector. 

Entomophagy has indeed recently gained a lot of attention as a potential contributor to the future of the sustainable food system. Depending on the source, between 1000 and 1900 edible insect species have been identified worldwide [4]. Globally, the most common insects consumed are beetles, caterpillars, bees, wasps, ants, grasshoppers, locusts, crickets, cicadas, leafhoppers, planthoppers, scale insects, true bugs, termites, dragonflies, and flies [4]. Edible insects are mainly consumed whole, processed in granular or paste form for other preparations, extracted insect proteins, and used in animal feed [4]. The main benefits of insects are related to the fact that they are a highly nutritious and healthy food source with high fat, protein, vitamin, fiber, mineral content, and a sustainable animal protein source [4]. Indeed, compared with other animal-based protein sources, insects request less water and space to be grown and emit fewer greenhouse gases. Moreover, insects show a much more efficient feed-conversation ratio than traditionally farmed animals. For example, “crickets are twice as efficient in converting feed to meat as chicken, at least four times more efficient than pigs, and 12 times more efficient than cattle” [4].

Insect farming could contribute to tackling the food waste challenge. Some insects can convert low-value products with low ecological footprints, such as unused co-products and organic side streams from the agri-food industries, into products with high protein content [5,6]. Insect farming has the potential to reuse at least a third of the food waste generated today in the food chain [7]. Moreover, insect frass, a by-product obtained from farmed insects, could be used as fertilizer and has recently been authorized by the European Commission [8]. Insect farming, therefore, has a limited impact on natural resources and could be an efficient and circular animal protein production system.

For these reasons, the EU has included insect breeding in the Farm to Fork Strategy and declared that insects could play a big role in developing a sustainable, resilient, and circular food system in the EU [9]. Insect farming could reduce imported food and feed products, shortening the food chain and diversifying the protein-based products available in the EU [8]. The commercialization of edible insects in the European Union is regulated by Regulation (EU) No. 2015/2283, which was then updated with new regulations. At present, three insect species, mealworms, locusts, and crickets, have been authorized in the EU [10]. The regulation includes both insects eaten whole and insects-based products, such as flour and snack [8]. As for the use of insects as feed, the Commission Regulation (EU) No. 2017/893 then updated with Regulation (EU) No. 2021/1372, which opened the use of processed animal protein (PAP) derived from seven insect species as feed for fish, pigs, and poultry [10]. According to the estimates of this market potential, the production of edible insects should be about 1 million tons in 2030, which will be transformed into 260,000 tons of insect-based foods with a turnover of 2 billion euros and 390 million consumers [6]. 

Edible insects are still an unusual foodstuff in European culture. Therefore, consumer rejection of entomophagy is one of the major barriers to the development of this market. Some scholars have focused their research on consumer acceptance of insects: Wendin & Nymberg (2021) [11] research this topic with a global vision whilst Ardoin & Prinyawiwatkul (2021) [12] and Dagevos (2021) [13] on the western countries. The recent work of Florença et al. (2022) [14] focuses on the difference between Western countries and traditional insect-eating countries. Regarding the European context, Mancini et al. (2019) [15] carried out the only systematic review on the theme while Kauppi et al. (2019) [16] focused on product characteristics and design interventions as adoption strategies. 

Over the years, there has been a steady increase in research in the field of edible insects, but there continues to be a scarcity of scientific contributions in the European context. Therefore, in order to increase knowledge on consumer perception, thus offering useful information on this potential market, a systematic review was conducted considering the latest progress in this research field. Specifically, this study is aimed, on the one hand, at defining the barriers and drivers of insect consumption in Europe and, on the other, at identifying the most promising strategies to convince European consumers to eat insects.

This systematic review is structured as follows: the methodology that carried out the research activity will be presented in Section 2. Section 3 is then dedicated to the main results emerging from the literature and replies to the first research question. Finally, the results of this systematic review will be discussed in Section 4, focusing on the second objective posed in this article (i.e., which are the most promising strategies to increase the reach of the European edible insect food market?). 

## 2. Materials and Methods

### 2.1. Choosing a Review Methodology

This systematic review was implemented according to the PRISMA (Preferred Reporting Items for Systematic Reviews and Meta-Analyses) methodology [17], as it has already been used by other authors recently reviewing factors influencing the consumer acceptability of alternative protein sources [18,19] or the relationship between food consumption and consumers’ environmental awareness [20,21]. To perform a systematic review within a replicable, scientific, and transparent approach it was decided to integrate the PRISMA guidelines with the model proposed by Tranfield et al. (2003) [22], as it has already been applied in other systematic reviews in the field of consumer preference evaluation [23]. The proposed method consists of a three-stage process: planning the review (Section 2.1 and Section 2.2), conducting the review (Section 2.3), and reporting and dissemination (Section 3). 

It was chosen to perform a systematic review instead of a meta-analysis because performing that type of study requires a high level of similarity among the methodologies applied across the various studies in terms of regression analysis, study design, samples, and context [24], leading to the forced exclusion of most of the selected publications. It was therefore decided not to limit the review to this type of study, thus including a good number of publications that apply qualitative, quantitative, and experimental methodologies to better represent the literature and main findings on the subject. 

### 2.2. Research Questions Definition 

Given the desirability of an increase in the consumption of insect-based proteins, the broader objective of this systematic review is that of exploring the main issues and current difficulties relating to the increase in the consumption of insects within consumer diets. Following an initial exploratory analysis of the literature on the subject, it was therefore decided to formulate two main research questions:

- Research Question 1 (RQ1): Among the variables analyzed in the literature, which appear to be barriers and which drivers for the consumption of insects in Europe?
- Research Question 2 (RQ2): Considering what emerged from the first research question, which are the most promising strategies to convince European consumers to eat insects?

The first research question will be covered in the results section (Section 3) while the second one will be explored in detail in the discussion (Section 4). 

### 2.3. Articles Selection Procedure

In order to find the most relevant peer-reviewed literature for the purpose of this review, and, therefore, to answer the research questions just mentioned, the following steps were initially followed: database selection, selection of keywords for the query, and eligibility criteria to be applied. 

First, the databases to be used for researching the scientific literature on the subject were decided. The authors choose mainly two databases: Scopus by Elsevier and Web of Science (WoS) by Thomson Reuters, as they are recognized today as being among the largest databases of abstracts and citations of peer-reviewed literature [19,25,26]. Therefore, they ensure the best coverage to find suitable publications covering the literature on the subject. As for the keywords used for the query, the authors chose to use the combination of words reported in Table 1. 

**Figure 1:**
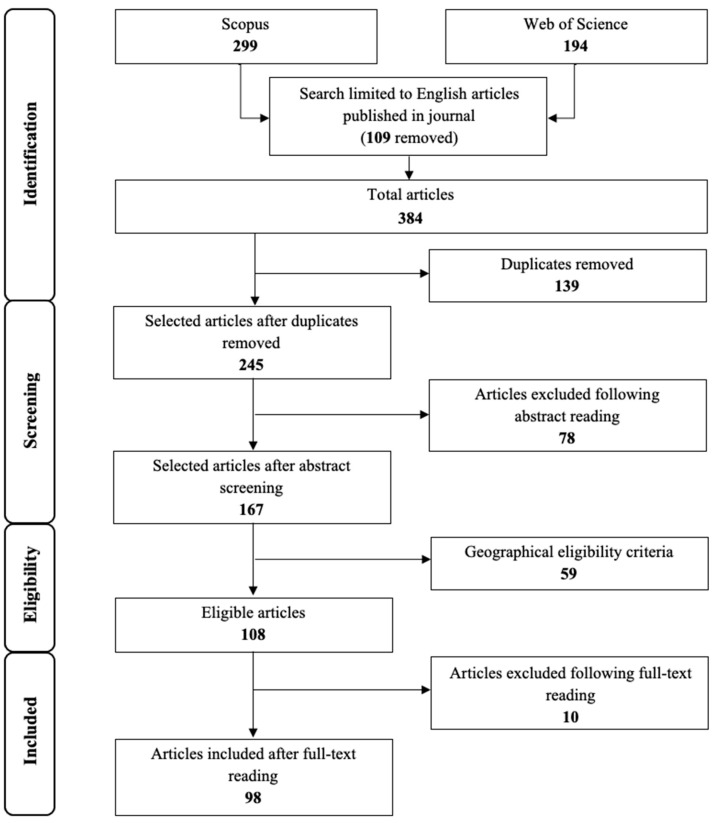
PRISMA flow diagram for the article selection process (numbers in bold indicate the number of papers for each stage of the systematic review).

The database search strategy was performed using the SPICE (Setting, Population, Intervention, Comparison, Evaluation) framework [27] as recently applied by other systematic reviews in this field of study [23,26]. The keywords specified in the table were then combined on Scopus and WoS using the operator “OR” between terms and the operator “AND” between different SPICE elements. As reported in the table, no term has been assigned to the SPICE “Intervention” element. Using the term “consumer” in combination with “insect food” in the search string already implied the argument of interest. Adding terms such as “acceptability” or “willingness to eat” terms resulted in too few articles: the mentioned combination of words is therefore assumed to be exhaustive for the purpose of this review. The exact search string used in Scopus is the following: TITLE-ABS-KEY (consumer) AND (TITLE-ABS-KEY (“insect as food”) OR TITLE-ABS-KEY (“insect food”) OR TITLE-ABS-KEY (“insect-based”)). The same research was performed in WoS using the following search string: (TI = (consumer) OR AB = (consumer) OR AK = (consumer)) AND ((TI = (“insect food”) OR AB = (“insect food”) OR AK = (“insect food”)) OR (TI = (“insect as food”) OR AB = (“insect as food”) OR AK = (“insect as food”)) OR (TI = (“insect-based”) OR AB = (“insect-based”) OR AK = (“insect-based”))). This activity was carried out on 21 November 2022. Figure 1 summarized the entire article selection process.

In the phase of “Identification”, 493 potentially selectable publications were detected, of which there were 299 of Scopus and 194 of WoS. Hence, the first set of eligibility criteria was used to filter these articles in order to remove publications that were not relevant. It was decided to keep only scientific peer-reviewed articles written in the English language and published in journals. Therefore, 109 publications were removed during this first phase, accounting for all non-English publications and contributions other than articles like reviews, book chapters, and conference proceedings. The inclusion and exclusion criteria used during the article selection procedure are listed in Table 2. After the application of the first two eligibility criteria, 139 duplicates were eliminated as they were present in the two databases. 

In the following “Screening” phase, with the application of the “during abstract screening” eligibility criteria, the goal was to eliminate the papers that were not relevant. Therefore, only the articles in which the main focus was the acceptability or the willingness to eat insects by consumers were kept. Of 245 eligible papers, 78 articles were eliminated as they focused on other aspects of the insect food supply chain that were not useful to answer the research questions posed in this review. 

**Figure 2:**
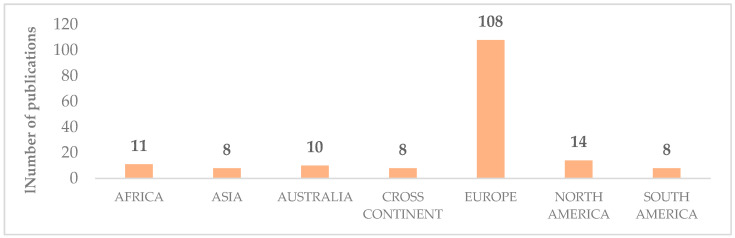
Geographical distribution of publications.

In the “Eligibility” phase, on the base of the information extracted during the abstract screening, the authors grouped the 167 selected articles according to the geographical area. As reported in Figure 2, which shows the geographical distribution of the selected publications, 65% of the articles refer to the European context. Therefore, the authors decided to focus the systemic review exclusively on Europe, excluding 59 papers that analyzed the topic in other countries. At the end of this phase, there were 108 papers for the full-text reading. 

This last phase then led to the further elimination of 10 articles that were not relevant to the objectives of this systematic review, leaving 98 articles included in the analysis presented in the next section. 

## 3. Results

### 3.1. Descriptive Statistics of Final Selected Papers

**Figure 3:**
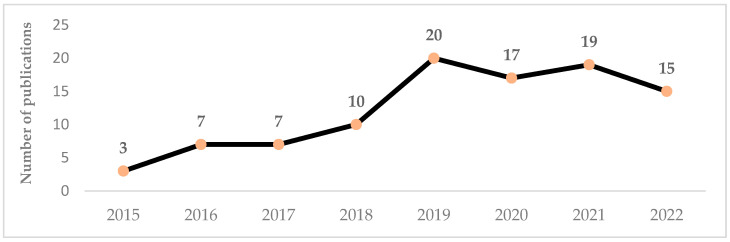
Distributions of articles per year of publication.

Before starting with the systematic literature review, there are some aspects to highlight that are useful for making a first contextualization of the 98 papers taken into consideration. Three-quarters of the articles were concentrated in the last four years and therefore demonstrate the growing interest of the international scientific community on this topic (Figure 3).

Regarding the sources, a total of 34 different journals were counted. Among these, only 8 journals counted for more than 2 publications. The journal that accounted for the largest number of publications is Food Quality and Preference (20 publications), followed by Journal of Insects as Food and Feed (10 publications), British Food Journal (9 publications), Foods (9 publications), Insects (6 publications), Appetite (4 publications), Food Research International (4 publications), and Sustainability (3 publications). 

**Figure 4:**
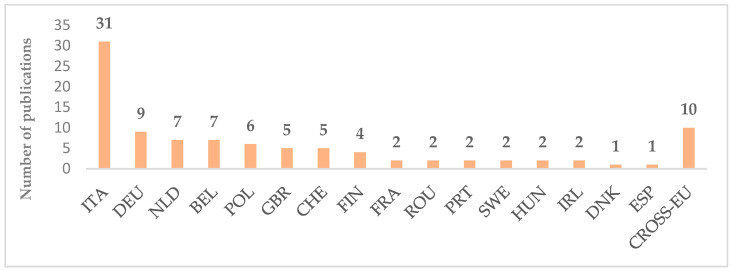
Geographical distributions of selected publications.

As for the geographic distribution of case study implementation, reported in Figure 4, the vast majority took place in Italy (31 publications), followed by Germany (9 publications), the Netherlands, and Belgium (7 publications). In total, 19 European Countries are involved in the analyzed publications, covering most of the European consumers. Among the selected articles, 88 presented a single-country study while 10 articles considered more than one European Country. 

Furthermore, other interesting features that emerge from a first summary analysis of the selected publications are related to the sample that was analyzed, the methodology and tools used to collect data, the methodology used to analyze the data, and the type of product taken into consideration. 

As for the sample analyzed to assess the acceptability of the insect as food, for most of the articles, the sample is made up of generic consumers or university students. There are some special cases, such as samples composed of specific population groups (young or older people) or samples composed of a panel of experts or people employed in the insect food sector. Different categories of people usually have different opinions on issues like food acceptability. Understanding these differences is therefore important if the goal is to understand the best strategy to increase the use of insects as food. 

The methodology used to collect the data in most cases was the survey. Furthermore, other techniques have also been employed. Some publications use interviews, focus groups, or images of edible insects to test the visual acceptability of these products. Then, a rather substantial group of articles uses experimental methods, such as tasting sessions, to text consumers’ opinions on entomophagy. 

Among the statistical methodologies for data processing, the most used is a regression analysis, followed by other techniques, such as univariate and multivariate statistics, factor and cluster analysis, ANOVA and ANCOVA analysis, the test of various statistical hypotheses, and qualitative analysis.

Finally, the issue has been addressed by scholars in a different way. Some research has investigated the level of acceptability of entomophagy without making any product distinction. Other research distinguishes between whole or processed insects, analyzing only one type or both. The most analyzed of the latter are burgers, bars, chips, biscuits, and bread. Furthermore, some studies analyze the difference in the level of acceptability of edible insects between those intended for human consumption and those intended for animal feed. The selected papers for the systematic review, together with the aforementioned features, are listed in Appendix A.

### 3.2. Drivers and Barriers to Entomophagy

On a general level, a very low willingness to try insect-based products (below 30%) can be seen in a significant part of the selected articles [28,29,30,31,32,33,34,35,36,37,38,39,40,41,42,43,44], but higher percentages also emerge [45,46,47,48,49,50]. Regardless, as it will be shown later in this section, the level of acceptability is different when it comes to processed products or insects used as feed. Given such a low level of acceptability, in order to understand how to increase the consumption of insects, it appears fundamental to analyze which of the variables analyzed in the literature seem to positively influence (drivers) and which negatively (barriers) the acceptability of insects as food (RQ1). This is particularly useful for understanding how the topic has been treated in order to build a strong basis for answering the main question posed in this systematic review (RQ2) (i.e., which are the best strategies to increase the reach of the edible insect food market?). The last research question will be analyzed in the discussion (Section 4). 

Among the selected papers, many different aspects were analyzed in order to assess the level of acceptability of insects as food. The first step followed was therefore to group the aspects that emerged from the literature into different macro-categories. This section is therefore divided as follows. The first subsection is related to the demographic characteristics of consumers, the second to the consumer psychological sphere, the third to the role of the sustainability argument, the fourth to the role of knowledge and information, the fifth to the role of culture and social norms, the sixth to the role of experience and familiarity with edible insects, and the seventh to the product features. Table 3 briefly explains the aspects analyzed in each subsection. 

However, this subdivision does not emerge clearly in the articles: there are indeed some studies that analyze only one of these categories of variables, but most of the publications analyze more aspects together. Since the subject has already been covered in the literature [11,12,13,14,15], here, the analysis is limited to the variables that are found to be significant in explaining the consumers’ opinion on edible insects. 

#### 3.2.1. Demographic Variables 

First, a large group of the selected publications considers the socio-demographic characteristics of consumers. The objective is that of studying the effect that these variables have on the level of acceptability and willingness to eat insects or the effect that these demographic features have on other variables that explains it. The most common demographic characteristics analyzed are related to gender, age, level/field of education, level of income, occupation, marital status, and place of residence (city or the countryside). Among these variables, those that were found to be significant relate to gender, age, level of education, and residence. Men are generally more inclined to accept insects than women, who in fact show higher levels of disgust [29,37,42,48,50,51,52,53,54,55]. Similarly, young people between the ages of 20 and 40 [31,32,36,44,49,56,57] and people with higher education [28,30,33,38,58,59] are more likely to be prone to entomophagy. Finally, some studies show greater acceptability of insects as food by the urban population compared to those who live in the countryside [35,49,59].

#### 3.2.2. Consumer Psychology 

A large group of papers then analyze different variables that can be indented among the personal/psychological/cognitive characteristics of consumers. The objective is that of understating the effect of these variables on the level of acceptability and willingness to eat or try insects, thus making use of mostly regression models. 

The first group of variables that can be included within the psychological sphere is related to the consumers’ perception and attitude toward insects. The main aspects analyzed are related to the level of food and insect neophobia (i.e., the reluctance to eat new foods) usually analyzed through the food neophobia scale, the level of disgust, or the perceived health risk implied by insects’ consumption. Likewise, some articles analyze the effect that curiosity can have on consumers’ attitudes toward trying new food (food neophilia). Subsequently, the main barriers to entomophagy that emerges among these variables are related to neophobia and disgust [30,32,34,35,42,54,55,57,58,60,61,62,63,64,65,66] and a perceived health risk associated with insect consumption [37,67,68]. On the other hand, curiosity about new experiences and new foods (i.e., food neophilia) [29,46,47,48] sometimes accounted by travel frequency [53] or a habit of eating in ethnic restaurants [28], positively influence the attitude towards insects. 

The second group of variables is then related to consumers’ eating habits and if they influence the intention to eat more sustainable protein such as insect-based food. The most common variables analyzed are related to the type of diet, frequency of meat consumption, meat reduction intention, food expenditure, and purchasing habits. The influence that these variables can have on the acceptability of the insects seems to be uncertain. In this regard, the study of [36] highlights how people who follow an omnivorous diet are more likely to try insects while conversely [69] points out that “Although 27% of participants were self-defined vegetarians, all but one were prepared to eat insects”. Some studies finally show that the intent to reduce meat consumption is a positive and significant predictor of the willingness to try other protein-based alternatives [29,58] while [31,53] does not find any significance of this predictor. 

#### 3.2.3. Sustainability 

Another topic that has been analyzed in the literature is related to whether consumers’ attitudes toward sustainability and environmental concerns influence the level of acceptability of entomophagy. The environmental benefits of insect-based protein have in fact often been used by public authorities to try to increase the consumption of insects, but the effect on consumers of this argument is still uncertain. In this regard, the role that environmental awareness may have on the consumer acceptability of insects as food does not emerge clearly within the literature. Some of the selected publications show that the positive attitude of consumers towards the environmental issue has a significant and positive influence on the willingness to try insect-based products [30,33,34,36,37,42,48,56,58,70,71,72,73]. On the contrary, although the influence of the environmental variable never appears negative, some publications find no significance in the consumers’ sustainability awareness in predicting the acceptability of entomophagy [35,38,55,64,74,75,76]. In this regard, as highlighted by [31,32,77], even when consumers have a strong propensity for environmental issues and sustainable diets, this does not translate into a higher level of acceptability and willingness to try insect-based products. Furthermore, the studies of [45,78] show that even when the environmental variable positively affects the acceptability, the effect is still much lower than other variables that negatively influence it, such as disgust and food neophobia.

#### 3.2.4. Knowledge and Information 

The next approach that emerged in the literature is related to the importance of the knowledge about the benefits of eating insects and the information received in order to increase their acceptability. Among these studies, some focus on the role of knowledge about the nutritional and environmental benefits while others use more experimental tools to study how consumers’ acceptability changes before and after receiving certain information. This type of research is particularly important to understand which issues consumers are most sensitive to in order to find the best strategies to increase the consumption of insects. What clearly emerges from the literature is the positive role that knowledge and information on the environmental benefits deriving from the consumption of insects could have on consumers’ opinions [46,76,79,80,81] and especially among those already sensitive to sustainability issues [82]. Furthermore, this positive effect is not limited to the environmental sphere: some studies that analyze the role of additional information around entomophagy [51,78,81,83,84,85,86] show that general knowledge, as well as single information about the nutritional, health, and environmental benefits of insect consumption, could play an important role in reducing perceived risk and increasing positive consumer perceptions. 

#### 3.2.5. Culture and Social Norms

Subsequently, another aspect considered in the literature is related to the social and cultural variables behind the acceptability of insects. The approach followed is that of testing the effect that subjective norms and social influence (i.e., the process by which one’s behavior is influenced by other people and social beliefs) or the irrational consumers’ behavior due to Western culture could have on willingness to eat insects. Several of the selected publications confirm the role that social influence has on consumers’ negative opinions of entomophagy [71,87,88]. While the study of Berger & Wyss (2020) [89] shows a link between perceptions of descriptive social norms and the willingness to consume insects, demonstrating that norms could indeed have a negative or positive influence on consumers eating intentions and behavior. Similarly, [90,91], through different experiments, highlight how social norm influences the level of disgust and what is internalized as normal food by consumers. Finally, the study of Menozzi et al. (2017) [52] includes the incompatibility with local food culture among the significant barriers to insect consumption. 

#### 3.2.6. Experience and Familiarity

Another important driver that emerges from the literature is the familiarity with the concept of entomophagy, accounted by previous knowledge [42,53] or previous insects’ consumption [31,37,38,55,60,66]. Furthermore, insect-tasting experiences could play a big role in increasing their acceptability [76,92,93,94], diminishing disgust and the idea that insects are non-edible [68], and decreasing food neophobia [95]. In this regard, the study of Zielińska et al. (2020) [50] highlights that 60% of the respondents who had previous insect-eating experiences rated the insect taste as good and very good. Furthermore, the percentage of approval of the products tested in the tasting sessions is generally quite high [95,96,97,98,99,100]. This demonstrates the fact that the greatest barriers to insect consumption are the social and psychological ones and that they can be knocked down with insect-eating experiences. 

#### 3.2.7. Product Features

Moving on to the product characteristics that emerge from the literature, various approaches have been used to analyze consumer preferences toward insect-based products. The publications that analyze product features usually make use of experimental methodologies, such as tasting sessions or food images to test consumers’ reactions and preferences toward different products. Among the most analyzed sensory attributes, there are the general liking of insect-based products, appearance, taste, visibility, odor, and consistency. Among these attributes, the main barrier to entomophagy that emerges from the literature is related to the visibility of the insects. What emerges is that the level of acceptability and willingness to try is higher for processed products compared to unprocessed products [31,35,40,45,50,51,52,54,55,75,94,101,102,103,104,105]. Likewise, products displaying images of actual insects on the packaging received a lower level of consumer acceptability [106,107]. A limited number of publications then focus on some product characteristics other than the sensory attributes. Among these, there are some papers that analyze the product design, packaging and labeling [41,108,109], and the price factor [49,58,110,111]. The effect of these features will be discussed in the next session. 

Finally, among the publications that focus on product characteristics, those that assess the level of acceptability of insects as feed for animals deserve a mention. Among these articles, the products considered are eggs [76,112,113], fish [70,114,115], and poultry [81,82]. Some other articles instead do not only investigate the acceptability of insects used as feed but compare the acceptability between human consumption and animal consumption of insects [30,32,38,39,40,45,80,93,102,116]. The level of consumer acceptability between the direct/indirect consumption of insects is very different, and it is therefore important to underline this product distinction. What emerges is clearly a higher level of acceptability for insects used as feed compared to that of human consumption, with percentages that in some cases are very high: 72% [113], 85% [46], and 90% [115]. Interestingly, the study of Spartano & Grasso (2021) [113] highlights that only 17% of respondents were aware of insects as a potential animal feed, demonstrating the fact that consumers generally are not aware of this possibility, but, as shown by the high level of acceptability, they seem ready to try these products. Furthermore, the results of La Barbera et al. (2021) [61] show that consumers express a different degree of acceptability for different insect feed-reared animals: poultry and fish are generally more accepted, thus showing greater potential for these food sectors.

## 4. Discussion

In this section, starting from the results that emerged from the analysis carried out in Section 3, it will be discussed the main research question (RQ2) posed in this systematic review. As previously specified, the first research question was addressed to provide a solid basis for analyzing the future potential of the edible insect market. Given that this kind of market has the peculiarity of making both environmental and business sense [101], it is useful to investigate the best strategies to break down the still-existing barriers to the consumption of edible insects. What emerges from the literature is a still very strong presence of consumers’ psychological barriers, such as disgust and food neophobia, which negatively affect consumers’ acceptability of edible insects. Indeed, the European insect market is still in an embryonic state. As a first step, the regulatory context has been developed, which has allowed insects to be brought back within the “novel foods” category, but the market still requires several barriers to be overcome before it can be considered fully developed. In this section, based on the consumer characteristics that emerged from the literature, the best strategies for overcoming these barriers will therefore be analyzed in order to further develop the insect market. First, some considerations will be made on the effectiveness of the sustainability argument in convincing consumers to include insects in their diet. Then, the fundamental role of the dissemination of knowledge around entomophagy to break down the psychological barriers of consumption will be discussed. Subsequently, some considerations will be made on the structural barriers and the importance of increasing the reach of this new market. Finally, some product development and market strategies will be analyzed.

The first useful aspect for the purposes of this review and which, to the authors’ knowledge, has not yet been systematically analyzed in the literature is that relating to the effectiveness of the sustainability argument in influencing consumers’ opinions on edible insects. As reported in the results section, the role of environmental awareness does not emerge clearly in the literature. The analysis of Simeone et al. (2022) [77] shows that high sustainability consciousness and willingness to eat insects could also not be correlated. Even consumers with the intention to reduce meat consumption are not always willing to replace such proteins with edible insects [31,53]. Moreover, among several sustainable protein options, insects are usually in the last place among consumers’ preferences [30,117]. Therefore, what emerges from the literature is that despite the importance of the environmental benefits of the insect market, sustainability is a weak argument to persuade consumers to try insects. Although consumers consider sustainability to be very important, immediate benefits for the self are usually more important than long-term benefits for the community in everyday decisions related to consumer choices [118]. Similarly, according to the analysis conducted by Berger et al. (2018) [74] “the marketing of edible insects with a strong focus on beneficial but distant goals such as health benefits or positive consequences for the environment is not very efficient”. Therefore, a switch to more immediate advertising strategies seems to be better for increasing the consumption of insect-based products. 

One possible explanation for the ineffectiveness of the sustainability argument could be that consumers have very little knowledge about the nutritional and environmental benefits related to edible insects [38]. Given the fundamental role that information can play in changing consumers’ opinion on entomophagy and in reducing the level of disgust [113,119], a first important suggestion to increase the reach of the edible insect market is that Public Authorities commit themselves more to the dissemination of knowledge. The most important information that consumers need to know is related to the environmental and nutritional benefits of this type of product, as well as more precise and scientifically based information on the risks deriving from the consumption of insects [101,120,121]. In this regard, the role that chefs [71,122], celebrities, high-profile environmental advocates, and even consumers themselves (peer-to-peer marketing) could have in providing reliable information and emotionally engaging consumers was also underlined [101,123]. 

Another aspect that emerges from the literature analysis and that could drive consumers’ opinion is the familiarity with edible insects [68,95]. To reduce the level of disgust and, consequently, the level of acceptability of insects by consumers, it therefore seems fundamental to increase the occasions in which consumers can try this type of product. The role of structural barriers indeed appears essential in explaining low levels of insect consumption [52,99,106]. The lack of eating opportunities or availability of products in restaurants and supermarkets [69,101,124] could be considered more important reasons for not having consumed insects before rather than disgust [63]. The possibility of finding this type of product in familiar places, such as restaurants and supermarkets, could increase consumer trust in these products [107]. Instead, given that the main current possibility of buying insects is that of online shops, only a small number of consumers are attracted, and the choice of products is limited due to the duration of the transport [71]. What seems to emerge from the literature is, therefore, that consumers’ disgust towards insects is also related to the fact that they are not yet widely available. The disgust barrier could, therefore, be partially brought down with the development of the market. Furthermore, additional information about how to cook and prepare insects, as well as recipes [71,122], are required to help consumers to add insects to their diet and increase the sense of familiarity with these products. 

Subsequently, from the literature emerges several product development strategies to break down the psychological barriers to consumption. The first very promising one could be that of focusing on products processed in a familiar form [45,55,99,102]. Proposing a product to which consumers are accustomed, and in which insects are not visible, could initially help to break down the barrier of disgust and increase future acceptability. Subsequently, to make sure that the consumption of insects is not a one-time experience but a repeated one, it is essential to develop products with good taste attributes [107,109]. However, another strategy emerges for those consumers that are more influenced by the curiosity and enthusiasm to try new foods [68,72,125,126]. For these consumers, it could be useful to develop more unusual products and advertisement strategies that focus on the exotic and traditional insect role of some cultures [69,124]. Focusing on products referred to as delicacies and distinctive of a traditional cuisine could, therefore, be an effective strategy for this market niche. Furthermore, as with the diffusion of sushi and other ethnic specialties, focusing on these product features could be effective in turning edible insects into a popular and diffuse food trend. 

Moreover, a strategy related to the packaging of the product is that of adopting a type of package that fosters the perception of a product made in a certified, standardized factory, and clearly states that the product contains insects [107]. This could help in increasing consumers’ trust in the safety and quality of products. Furthermore, informative packaging that shows the benefits of edible insects may be another useful source of information to break down the barriers to consumption [68]. 

Finally, from the literature emerges that different market sections could coexist based on price differentiations. Some consumers express a willingness to pay for insect-based products that are very low [58,93,109] while others seem to be willing to pay for a premium product [71,113]. In this early market stage, the strategy of focusing on consumers willing to pay more for a high-quality product seems more promising [110]. Initially offering products with low prices could in fact lead consumers to perceive poor quality [109]. Subsequently, the development of the market together with the increase in consumer familiarity with edible insects will permit lower prices [101] and, therefore, a market opening to broader segments.

## 5. Conclusions

The process of reviewing the scientific literature on European consumers’ opinions on entomophagy has been useful for describing the current state-of-the-art and, therefore, for understanding the main challenges to the development of a mainstream edible insect market. This is particularly useful for outlining possible paths for further research and for contributing to increasing knowledge about the more efficient market strategy and policy development to increase the consumption of edible insects in Europe. This systematic review therefore helped advance existing research on the consumer side of the edible insect market. This seems essential given the embryonic stage of this market in Europe.

In particular, the strategies that seem most promising for breaking down barriers to insect consumption were highlighted. The first strategy that emerges from the literature is to increase the general knowledge of entomophagy and the environmental and nutritional benefits of insect eating. This is useful both to convince consumers who are more sensitive to these issues and to break down the still-existing prejudices related to the fact that insects are not considered edible food. This information can come from various sources. Some channels are considered more trusted by consumers, but what emerges is that public authorities, producers, the scientific community, as well as prominent personalities within society can all be helpful together in increasing knowledge. 

Another major barrier that has emerged from the literature concerns consumer familiarity with entomophagy. In fact, consumers need to get used to the idea that insects are edible before even thinking about including them in their diets. Insects must enter the daily life of consumers. The increase in the occasions in which these products can be tasted, their availability on the shelves of supermarkets and restaurants, and their inclusion in famous cooking programs can all be successful strategies for approaching and intriguing consumers to try insects for the first time. Expanding the market reach of edible insects could therefore be an effective strategy to break down barriers to consumption. Another aspect that emerged from this systematic review is the fact that the sustainability argument alone does not seem to be a very effective strategy to convince consumers to eat insects. Consumers’ opinions on environmental issues are indeed very important and could play a role in this challenge. However, the priority must be given to the dissemination of the knowledge of these benefits. Furthermore, a switch to advertising strategies that focus on the immediate benefits of the products, such as their nutritional value and taste attributes, would be better to promote the consumption of insect-based products. Finally, from the literature, it was possible to retrieve suggestions on the product characteristics preferred by consumers. This can be useful information for producers in order to develop more effective products and marketing strategies to increase market reach. 

This paper does, however, have some limitations. First, the conclusions were drawn based on studies that take into consideration only a limited sample of the European population. In many cases, the sample consisted of university students, who are usually more open about these topics. The opinions of all European consumers may therefore differ from those reported here. Consumers not sensitive to any of the strategies highlighted by this review could, indeed, always exist. Furthermore, the consumer side is only one of the aspects that need to be investigated to increase the consumption of insects. Although the barriers to consumption are mainly constituted by consumers’ disgust towards insects, other aspects also deserve to be investigated. The European regulatory context certainly affects the development of the market. Future research on the subject is, therefore, certainly useful. Similarly, taking the production potential and difficulties into account seems equally important. Finally, given that the market is not yet widely developed, it is essential to further research consumer opinions on the subject. A suggestion might be to conduct a meta-analysis of the articles that make use of a regression model. This type of analysis has never been used to analyze consumers’ opinions on entomophagy, but it is very often used in studies on consumer preferences. It would therefore be a useful tool for further analyzing the effect of the variables that emerged in this systematic review and would probably allow more precise suggestions to be made.

## Figures and Tables

**Table 1:** SPICE framework used for the database search strategy.

SPICE Element	Search Terms Assigned	Reason
*Setting*—where?	No term assigned	The interests of the review include all contexts
*Population*—for whom?	“Consumer”	To limit the information on consumers
*Intervention*—what?	No term assigned	Adding terms in this field yielded narrow results.
*Comparison*—compared with what?	No term assigned	Not interested in comparison with different products
*Evaluation*—with what result?	“Insect as food” “Insect food” “Insect-based”	The outcomes of interest are consumers’ acceptability and willingness to try insects as food

**Table 2:** Eligibility criteria.

Inclusion Criteria	Exclusion Criteria
*Before abstract screening:* - English language - Peer reviewed journal articles	*Before abstract screening:* - Other languages - Reviews, book chapters, proceeding articles
*During abstract screening:* - Focus on consumers acceptability or willingness to try/consume/pay insects European context	*During abstract screening:* - Focus on other aspects of the insect food supply chain-Different geographical context

**Table 3:** Summary of aspects analyzed in this section.

Section	Title	Description
Section 3.2.1	Demographic Variables	Which demographic variables shows an effect on the level of acceptability of insects
Section 3.2.2	Consumer Psychology	Variables included in this section comprise food neophobia, disgust, perceived risk, curiosity, and type of diet.
Section 3.2.3	Sustainability	Whether consumers’ attitudes toward sustainability and environmental concerns influence the level of acceptability
Section 3.2.4	Knowledge and Information	The role of consumers’ knowledge about the benefits of insects and of different information received on the level of acceptability
Section 3.2.5	Culture and Social Norms	The role of European culture, subjective norms, and social influence on the level of acceptability
Section 3.2.6	Experience and Familiarity	The role of previous insects’ consumption and familiarity with the concept of edible insect on the level of acceptability
Section 3.2.7	Product Features	Aspects analyzed in this section comprise sensory attributes of insects-based products, insects’ visibility, and insects as feed

## Data Availability

Data is contained within the article or supplementary material.

## Appendix A

Appendix A lists the articles selected for the systematic review together with some features.

**Reference**	**Area**	**Sample**	**Data Collection**	**Data Analysis**	**Type of Product**
Arena et al. (2020) [79]	ITA	Consumers	Survey	Regression	No distinction
Baldi et al. (2022) [70]	ITA	Consumers	Survey	Factor analysis/Regression (Rasch model)	Feed (Fish)
Balzan et al. (2015) [71]	ITA	Consumers	Focus group	Qualitative analysis	Both types
Barsics et al. (2017) [83]	BEL	University students	Tasting/Information	Regression (GLM model)	Processed (Bread)
Berger & Wyss (2020) [89]	CHE	University students	Experimental	Regression	No distinction
Berger et al. (2018) [110]	DEU	Consumers	Experimental	Regression	Both types
Berger et al. (2018) [74]	DEU	Consumers	Survey	Regression (OLS model/Probit model)	No distinction
Berger et al. (2019) [120]	CHE	University students/Consumers	Survey (Pictures)/Tasting	Regression	Processed
Bruckdorfer & Büttner (2022) [106]	DEU	University students	Survey/Tasting	Regression	Processed (Bars)
Caparros Megido et al. (2016) [95]	BEL	University students	Survey/Tasting	Regression (GLM model)	Processed (Burger)
Cavallo & Materia (2018) [75]	ITA	Millennials	Survey (Pictures)	Regression	Both types
Cicatiello et al. (2016) [28]	ITA	Consumers	Survey	Regression	No distinction
Cicatiello et al. (2020) [96]	ITA	University students	Survey/Tasting	ANCOVA	Both types
Collins et al. (2019) [101]	GBR/FRA	Children/Adults	Survey	Regression	Both types
Detilleux et al. (2020) [92]	BEL	Minors	Survey/Tasting	Regression (GLM model)	Processed (Falafel)
Fasanelli et al. (2020) [87]	ITA	Millennials/Gen Z	Survey/Tasting	Multiple statistical analysis	No distinction
Fischer & Steenbekkers (2018) [60]	NLD	University students	Survey	Regression	Whole insects
Florença et al. (2021) [51]	PRT	Consumers	Survey	Regression (CRT Model)	No distinction
Gallen et al. (2022) [108]	FRA	Consumers	Experimental		Processed
Gere et al. (2017) [29]	HUN	Consumers	Survey	Regression	No distinction
Grasso et al. (2019) [30]	UK/NLD/POL/ESP/FIN	Old consumers	Survey	Regression	No distinction
Halonen et al. (2022) [102]	FIN	Consumers/Experts	Survey/Interview	Descriptive statistics	Both types/Feed
Hartmann et al. (2018) [123]	CHE	Consumers	Experimental	Descriptive statistics/ANOVA	Processed
Herbert & Beacom (2021) [109]	IRL	Consumers	Survey/Focus group	Regression	Processed (Snack)
House (2016) [69]	NLD	Consumers who have already eaten insects	Interview	Descriptive statistics	Processed
Iannuzzi et al. (2019) [88]	ITA	Consumers	Survey	Descriptive statistics	Processed (Pizza)
Isaac Ankamah-Yeboah et al. (2018) [114]	DEU	Consumers	Survey (Pictures)	Regression (MNL model)/LC MODEL	Feed (Fish)
Iseppi et al. (2021) [93]	ITA	Consumers	Survey	Regression (Rasch Model)	Both types/Feed
Kane & Dermiki (2021) [45]	IRL	University students	Survey	Descriptive statistics/Test	Both types/Feed
Khalil et al. (2021) [33]	ESP	Consumers	Survey	Regression (MNL model)	Processed (Yogurt and Jam)
Koch et al. (2021) [90]	NLD	Consumers/Students/Participants in a health fair	Experimental	Descriptive statistics/Test	Both types
Kornher et al. (2019) [58]	DEU	Consumers	Survey (Pictures)	Conditional logit latent class model	No distinction
Kostecka et al. (2017) [80]	POL	Consumers	Survey	Descriptive statistics	Both types/Feed
La Barbera et al. (2018) [61]	ITA/DNK	University students	Survey/Tasting	PLS Path Modelling	No distinction
La Barbera et al. (2021) [67]	ITA	Consumers	Survey	Regression	No distinction/Feed
Lammers et al. (2019) [31]	DEU	Consumers	Survey	Regression	Both types
Laureati et al. (2016) [32]	ITA	Consumers/University students/University staff	Survey (Pictures)	Regression (GLM model)	Both types/Feed
Le Goff & Delarue (2017) [62]	FRA	Consumers	Tasting	ANOVA	Processed (Chips)
Lippi et al. (2021) [112]	ITA	Consumers	Survey	Regression (OLS model)/Cluster analysis	Feed (Eggs)
Lombardi et al. (2019) [63]	ITA	University students	Survey	Regression	Processed
Lorini et al. (2021) [119]	ITA	University students	Survey	Descriptive statistics/test	No distinction
Lunden et al. (2020) [117]	FIN	Consumers	Survey	Descriptive statistics/Test	No distinction
Mancini et al. (2019) [84]	ITA	Consumers	Survey/Tasting	Regression	Both types
Mandolesi et al. (2022) [104]	ITA	Experts/University students/University staff	Survey (Pictures)	Q Analysis	Both types
Marin et al. (2021) [122]	ITA	Consumers	Survey	Regression	No distinction
Martins et al. (2022) [103]	PRT/ROU/SRB	Consumers	Survey	Regression (SEM model)	Both types
Menozzi et al. (2017) [52]	ITA	University students	Survey	TBP—Regression	Processed (Biscuits)
Menozzi et al. (2021) [81]	ITA	Consumers	Survey/Information	Descriptive statistics	Feed (Poultry)
Milani & Jacomuzzi (2020) [121]	ITA	Young Consumers (20–25 years old)	Focus group	Qualitative content analysis	No distinction
Modlinska et al. (2020) [64]	POL	Consumers	Tasting/Survey/interview	Descriptive statistics/ANOVA/ANCOVA	Processed
Modlinska et al. (2021) [53]	POL	Consumers	Survey	Regression	No distinction
Moruzzo et al. (2021) [65]	ITA	Consumers	Survey	Regression (Logit model)	No distinction
Narango-Guevara et al. (2021) [46]	NLD/DEU	University students	Survey (Pictures)	Regression	No distinction/Feed
Niva & Vainio (2021) [34]	FIN	Consumers	Survey	Regression (MNL model)/LC MODEL	No distinction
Nyberg et al. (2020) [72]	SWE	Consumers	Survey/Workshop discussion	Descriptive statistics	Both types
Onwezen et al. (2019) [116]	NLD	Consumers	Survey	Regression	Both types/Feed
Orkusz et al. (2020) [35]	POL	University students	Survey/Tasting	Regression	Both types
Orsi et al. (2019) [36]	DEU	Consumers	Survey	Regression	Both types
Padulo et al. (2022) [68]	ITA	Consumers	Survey/Tasting	Regression	Processed (Bars)
Palmieri et al. (2019) [37]	ITA	Consumers	Survey	Regression	No distinction
Penedo et al. (2022) [47]	CHE	University students	Survey	Descriptive statistics	Both types
Petruscu-Mag et al. (2022) [56]	ROU	Consumers	Survey/Interview	Regression	Processed
Piha et al. (2018) [66]	FIN/SWE/CZE/DEU	Consumers	Survey	Regression (SEM model)	No distinction
Poortvliet et al. (2019) [125]	NLD	Consumers	Survey (Pictures)	Descriptive statistics/ANOVA	Processed
Popoff et al. (2017) [115]	GBR/SWE	Consumers/Stakeholders	Survey/Interview	Descriptive statistics	Feed (Fish)
Powell et al. (2019) [111]	GBR	Consumers	Survey (Pictures)	Descriptive statistics	Processed
Reverberi (2021) [107]	SWE	Producers	Interview	Qualitative analysis	Both types
Ribeiro et al. (2022a) [38]	PRT/NOR	Consumers	Survey	Regression	Both types/Feed
Ribeiro et al. (2022b) [97]	PRT	Consumers	Tasting	Descriptive statistics/Test	Processed (Bars)
Roma et al. (2020) [39]	ITA	Consumers	Survey	Rough set model	Both types/Feed
Rumpold et al. (2019) [85]	DEU	Consumers (Participants in a science fair)	Interview/Tasting/Information	Descriptive statistics/Test	Both types
Russel & Knott (2021) [91]	GBR	Consumers	Survey	Regression	Both types
Schäufele et al. (2019) [54]	DEU	Consumers	Survey (Pictures)	Regression	Both types
Schlup & Brunner (2018) [55]	CHE	Consumers	Survey	Regression (Tobit model)	Both types
Schouteten et al. (2016) [86]	BEL	University students	Tasting/Information	Descriptive statistics/test	Processed (Burger)
Sidali et al. (2019) [124]	ITA	University students	Survey	Regression (SEM model)	Both types
Simeone et al. (2022) [77]	ITA	Consumers	Survey	Regression (Probit model)	No distinction
Simion et al. (2019) [40]	ROU	University students	Survey	Regression	Both types/Feed
Smarzyński et al. (2019) [98]	POL	Consumers	Tasting	Descriptive statistics/Test	Processed (Patè)
Sogari (2015) [73]	ITA	Consumers	Survey/Tasting	Qualitative content analysis	No distinction
Sogari et al. (2018) [94]	ITA	Consumers	Tasting	Descriptive statistics	Both types
Sogari et al. (2019) [57]	ITA	University students/Staff	Survey/Tasting	Regression (SEM Model)	Both types
Sogari et al. (2022) [82]	ITA	Consumers	Survey/Information	Regression (SEM model)	Feed (Poultry)
Spartano & Grasso (2021) [113]	GBR	Consumers	Survey	Regression (Binary model/Tobit model)	Feed (Eggs)
Spartano et al. (2021) [76]	GBR	Consumers	Focus group	Qualitative analysis	Feed (Eggs)
Stone et al. (2022) [126]	GBR	Consumers	Survey (Pictures)	Regression (Mixed model)	No distinction
Szendrő et al. (2020) [59]	HUN	Consumers	Survey	Regression	No distinction
Tan et al. (2016) [105]	NLD	Consumers	Survey (Pictures)	Regression (Hierarchical model)	Both types
Tan et al. (2017) [99]	NLD	Consumers	Survey/Tasting	Regression	Both types
Tucillo et al. (2020) [48]	ITA	Consumers	Survey/Tasting	Regression/Cluster analysis	Both types
Tzompa-sosa et al. (2022) [100]	BEL	Consumers	Tasting	PCA analysis	Processed (Fries)
Van Thielen et al. (2019) [41]	BEL	Consumers	Survey	Descriptive statistics	Processed
Vartiainen et al. (2020) [49]	FIN	Consumers	Survey	Regression—Cluster analysis	No distinction
Verbeke (2015) [42]	BEL	Consumers	Survey	Regression	No distinction
Verneau et al. (2016) [78]	ITA/DNK	University students	Tasting/Information	Regression	Processed (Bars)
Verneau et al. (2020) [43]	ITA/DNK	University students	Survey	Factor analysis/Cluster analysis	No distinction
Videbaek et al. (2020) [44]	DNK	Consumers	Survey	Regression (Hierarchical model)	Both types
Zielińska et al. (2020) [50]	POL	Consumers	Survey	Descriptive statistics/Test	Both types

## References

[1] FAO (2017). The Future of Food and Agriculture—Trends and Challenges. https://www.fao.org/3/i6583e/i6583e.pdf.

[2] Henchion M., Hayes M., Mullen A.M., Fenelon M., Tiwari B. (2017). Future Protein Supply and Demand: Strategies and Factors Influencing a Sustainable Equilibrium. Foods.

[3] Godfray H.C.J., Beddington J.R., Crute I.R., Haddad L., Lawrence D., Muir J.F., Pretty J., Robinson S., Thomas S.M., Toulmin C. (2010). Food Security: The Challenge of Feeding 9 Billion People. Science.

[4] FAO (2013). Edible Insects. Future Prospects for Food and Feed Security. https://www.fao.org/3/i3253e/i3253e.pdf.

[5] IPIFF (2018). IPIFF Contribution on the Development of a European Protein Plan. https://ipiff.org/wp-content/uploads/2018/10/IPIFF-contribution-on-the-development-of-a-European-Protein-Plan-28-09-2018.pdf.

[6] IPIFF (2019). The European Insect Sector Today: Challenges, Opportunities and Regulatory Landscape. IPIFF Vision Paper on the Future of the Insect Sector towards 2030. https://ipiff.org/wp-content/uploads/2019/12/2019IPIFF_VisionPaper_updated.pdf.

[7] IPIFF (2020). The Contribution of the European Insect Sector to Improving Sustainability from ‘Farm to Fork’. https://ipiff.org/wp-content/uploads/2020/02/18-02-2020-IPIFF-Position-Paper-on-the-F2F.pdf.

[8] Delgado L., Garino C., Moreno F.J., Zagon J., Broll H. (2022). Sustainable Food Systems: EU Regulatory Framework and Contribution of Insects to the Farm-To-Fork Strategy. Food Rev. Int..

[9] IPIFF (2020). The Insect Sector Milestones towards Sustainable Food Supply Chains. https://ipiff.org/wp-content/uploads/2020/05/IPIFF-RegulatoryBrochure-update07-2020-1.pdf.

[10] Formici G., Scaffardi L., Formici G. (2022). Legislative and Judicial Challenges on Insects for Human Consumption: From Member States to the EU, Passing Through the Court of Justice of the EU. Novel Foods and Edible Insects in the European Union.

[11] Wendin K.M., Nyberg M.E. (2021). Factors Influencing Consumer Perception and Acceptability of Insect-Based Foods. Curr. Opin. Food Sci..

[12] Ardoin R., Prinyawiwatkul W. (2021). Consumer Perceptions of Insect Consumption: A Review of Western Research since 2015. Int. J. Food Sci. Technol..

[13] Dagevos H. (2021). A Literature Review of Consumer Research on Edible Insects: Recent Evidence and New Vistas from 2019 Studies. J. Insects Food Feed..

[14] Florença S.G., Guiné R.P.F., Gonçalves F.J.A., Barroca M.J., Ferreira M., Costa C.A., Correia P.M.R., Cardoso A.P., Campos S., Anjos O. (2022). The Motivations for Consumption of Edible Insects: A Systematic Review. Foods.

[15] Mancini S., Moruzzo R., Riccioli F., Paci G. (2019). European Consumers’ Readiness to Adopt Insects as Food. A Review. Food Res. Int..

[16] Kauppi S.-M., Pettersen I.N., Boks C. (2019). Consumer Acceptance of Edible Insects and Design Interventions as Adoption Strategy. Int. J. Food Des..

[17] Moher D., Shamseer L., Clarke M., Ghersi D., Liberati A., Petticrew M., Shekelle P., Stewart L.A., PRISMA-P Group (2015). Preferred Reporting Items for Systematic Review and Meta-Analysis Protocols (PRISMA-P) 2015 Statement. Syst. Rev..

[18] Kröger T., Dupont J., Büsing L., Fiebelkorn F. (2022). Acceptance of Insect-Based Food Products in Western Societies: A Systematic Review. Front. Nutr..

[19] Pakseresht A., Ahmadi Kaliji S., Canavari M. (2022). Review of Factors Affecting Consumer Acceptance of Cultured Meat. Appetite.

[20] Sánchez L.A., Roa-Díaz Z.M., Gamba M., Grisotto G., Londoño A.M.M., Mantilla-Uribe B.P., Méndez A.Y.R., Ballesteros M., Kopp-Heim D., Minder B. (2021). What Influences the Sustainable Food Consumption Behaviours of University Students? A Systematic Review. Int. J. Public Health.

[21] Sanchez-Sabate R., Sabaté J. (2019). Consumer Attitudes towards Environmental Concerns of Meat Consumption: A Systematic Review. Int. J. Environ. Res. Public Health.

[22] Tranfield D., Denyer D., Smart P. (2003). Towards a Methodology for Developing Evidence- Informed Management Knowledge by Means of Systematic Review. Br. J. Manag..

[23] Stiletto A., Trestini S. (2021). Factors behind Consumers’ Choices for Healthy Fruits: A Review of Pomegranate and Its Food Derivatives. Agric. Food Econ..

[24] Leonidou E., Christofi M., Vrontis D., Thrassou A. (2020). An Integrative Framework of Stakeholder Engagement for Innovation Management and Entrepreneurship Development. J. Bus. Res..

[25] Burnham J. (2006). Scopus Database: A Review. Biomed. Digit. Libr..

[26] Cantillo J., Martín J.C., Román C. (2020). Discrete Choice Experiments in the Analysis of Consumers’ Preferences for Finfish Products: A Systematic Literature Review. Food Qual. Prefer..

[27] Cleyle S., Booth A. (2006). Clear and Present Questions: Formulating Questions for Evidence Based Practice. Libr. Hi Tech.

[28] Cicatiello C., De Rosa B., Franco S., Lacetera N. (2016). Consumer Approach to Insects as Food: Barriers and Potential for Consumption in Italy. Br. Food J..

[29] Gere A., Székely G., Kovács S., Kókai Z., Sipos L. (2017). Readiness to Adopt Insects in Hungary: A Case Study. Food Qual. Prefer..

[30] Grasso A.C., Hung Y., Olthof M.R., Verbeke W., Brouwer I.A. (2019). Older Consumers’ Readiness to Accept Alternative, More Sustainable Protein Sources in the European Union. Nutrients.

[31] Lammers P., Ullmann L.M., Fiebelkorn F. (2019). Acceptance of Insects as Food in Germany: Is It about Sensation Seeking, Sustainability Consciousness, or Food Disgust?. Food Qual. Prefer..

[32] Laureati M., Proserpio C., Jucker C., Savoldelli S. (2016). New Sustainable Protein Sources: Consumers’ Willingness to Adopt Insects as Feed and Food. Ital. J. Food Sci..

[33] Khalil R., Kallas Z., Haddarah A., El Omar F., Pujolà M. (2021). Impact of COVID-19 Pandemic on Willingness to Consume Insect-Based Food Products in Catalonia. Foods.

[34] Niva M., Vainio A. (2021). Towards More Environmentally Sustainable Diets? Changes in the Consumption of Beef and Plant- and Insect-Based Protein Products in Consumer Groups in Finland. Meat Sci..

[35] Orkusz A., Wolańska W., Harasym J., Piwowar A., Kapelko M. (2020). Consumers’ Attitudes Facing Entomophagy: Polish Case Perspectives. Int. J. Environ. Res. Public Health.

[36] Orsi L., Voege L.L., Stranieri S. (2019). Eating edible insects as sustainable food? Exploring the determinants of consumer acceptance in Germany. Food Res. Int..

[37] Palmieri N., Perito M.A., Macrì M.C., Lupi C. (2019). Exploring Consumers’ Willingness to Eat Insects in Italy. Br. Food J..

[38] Ribeiro J.C., Gonçalves A.T.S., Moura A.P., Varela P., Cunha L.M. (2022). Insects as Food and Feed in Portugal and Norway—Cross-Cultural Comparison of Determinants of Acceptance. Food Qual. Prefer..

[39] Roma R., Palmisano G.O., De Boni A. (2020). Insects as Novel Food: A Consumer Attitude Analysis through the Dominance-Based Rough Set Approach. Foods.

[40] Simion V.E., Bucea-Manea R., Adriana A., Dourado Martins O.M., Sekovska B., Dijmărescu I. (2019). Entomofagy—a Viable Solution for Supporting Food Security. Amfiteatru Econ..

[41] Van Thielen L., Vermuyten S., Storms B., Rumpold B., Van Campenhout L. (2019). Consumer Acceptance of Foods Containing Edible Insects in Belgium Two Years after Their Introduction to the Market. J. Insects Food Feed..

[42] Verbeke W. (2015). Profiling Consumers Who Are Ready to Adopt Insects as a Meat Substitute in a Western Society. Food Qual. Prefer..

[43] Verneau F., Barbera F.L., Amato M., Riverso R., Grunert K.G. (2020). Assessing the Role of Food Related Lifestyle in Predicting Intention towards Edible Insects. Insects.

[44] Videbaek P.N., Grunert K.G. (2020). Disgusting or Delicious? Examining Attitudinal Ambivalence towards Entomophagy among Danish Consumers. Food Qual. Prefer..

[45] Kane B., Dermiki M. (2021). Factors and Conditions Influencing the Willingness of Irish Consumers to Try Insects: A Pilot Study. Ir. J. Agric. Food Res..

[46] Naranjo-Guevara N., Fanter M., Conconi A.M., Floto-Stammen S. (2021). Consumer Acceptance among Dutch and German Students of Insects in Feed and Food. Food Sci. Nutr..

[47] Penedo A.O., Bucher Della Torre S., Götze F., Brunner T.A., Brück W.M. (2022). The Consumption of Insects in Switzerland: University-Based Perspectives of Entomophagy. Foods.

[48] Tuccillo F., Marino M.G., Torri L. (2020). Italian Consumers’ Attitudes towards Entomophagy: Influence of Human Factors and Properties of Insects and Insect-Based Food. Food Res. Int..

[49] Vartiainen O., Elorinne A.L., Niva M., Väisänen P. (2020). Finnish Consumers’ Intentions to Consume Insect-Based Foods. J. Insects Food Feed..

[50] Zielińska E., Zieliński D., Karaś M., Jakubczyk A. (2020). Exploration of Consumer Acceptance of Insects as Food in Poland. J. Insects Food Feed..

[51] Florença S.G., Correia P.M.R., Costa C.A., Guiné R.P.F. (2021). Edible Insects: Preliminary Study about Perceptions, Attitudes, and Knowledge on a Sample of Portuguese Citizens. Foods.

[52] Menozzi D., Sogari G., Veneziani M., Simoni E., Mora C. (2017). Eating Novel Foods: An Application of the Theory of Planned Behaviour to Predict the Consumption of an Insect- Based Product. Food Qual. Prefer..

[53] Modlinska K., Adamczyk D., Maison D., Goncikowska K., Pisula W. (2021). Relationship between Acceptance of Insects as an Alternative to Meat and Willingness to Consume Insect-Based Food—A Study on a Representative Sample of the Polish Population. Foods.

[54] Schäufele I., Barrera Albores E., Hamm U. (2019). The Role of Species for the Acceptance of Edible Insects: Evidence from a Consumer Survey. Br. Food J..

[55] Schlup Y., Brunner T. (2018). Prospects for Insects as Food in Switzerland: A Tobit Regression. Food Qual. Prefer..

[56] Petrescu-Mag R.M., Rastegari Kopaei H., Petrescu D.C. (2022). Consumers’ Acceptance of the First Novel Insect Food Approved in the European Union: Predictors of Yellow Mealworm Chips Consumption. Food Sci. Nutr..

[57] Sogari G., Menozzi D., Mora C. (2019). The Food Neophobia Scale and Young Adults’ Intention to Eat Insect Products. Int. J. Consum. Stud..

[58] Kornher L., Schellhorn M., Vetter S. (2019). Disgusting or Innovative-Consumer Willingness to Pay for Insect Based Burger Patties in Germany. Sustainability.

[59] Szendrö K., Tóth K., Nagy M.Z. (2020). Opinions on Insect Consumption in Hungary. Foods.

[60] Fischer A.R.H., Steenbekkers L.P.A. (2018). All Insects Are Equal, but Some Insects Are More Equal than Others. Br. Food J..

[61] La Barbera F., Verneau F., Amato M., Grunert K. (2018). Understanding Westerners’ Disgust for the Eating of Insects: The Role of Food Neophobia and Implicit Associations. Food Qual. Prefer..

[62] Le Goff G., Delarue J. (2017). Non-Verbal Evaluation of Acceptance of Insect-Based Products Using a Simple and Holistic Analysis of Facial Expressions. Food Qual. Prefer..

[63] Lombardi A., Vecchio R., Borrello M., Caracciolo F., Cembalo L. (2019). Willingness to Pay for Insect-Based Food: The Role of Information and Carrier. Food Qual. Prefer..

[64] Modlinska K., Adamczyk D., Goncikowska K., Maison D., Pisula W. (2020). The Effect of Labelling and Visual Properties on the Acceptance of Foods Containing Insects. Nutrients.

[65] Moruzzo R., Mancini S., Boncinelli F., Riccioli F. (2021). Exploring the Acceptance of Entomophagy: A Survey of Italian Consumers. Insects.

[66] Piha S., Pohjanheimo T., Lähteenmäki-Uutela A., Křečková Z., Otterbring T. (2018). The Effects of Consumer Knowledge on the Willingness to Buy Insect Food: An Exploratory Cross- Regional Study in Northern and Central Europe. Food Qual. Prefer..

[67] La Barbera F., Amato M., Fasanelli R., Verneau F. (2021). Perceived Risk of Insect-Based Foods: An Assessment of the Entomophagy Attitude Questionnaire Predictive Validity. Insects.

[68] Padulo C., Carlucci L., Balsamo M., Fairfield B. (2022). A Dynamic Hop to Cricket Consumption: Factors Influencing Willingness to Try Insect based Food. J. Insects Food Feed..

[69] House J. (2016). Consumer Acceptance of Insect-Based Foods in the Netherlands: Academic and Commercial Implications. Appetite.

[70] Baldi L., Mancuso T., Peri M., Gasco L., Trentinaglia M.T. (2022). Consumer Attitude and Acceptance toward Fish Fed with Insects: A Focus on the New Generations. J. Insects Food Feed..

[71] Balzan S., Fasolato L., Maniero S., Novelli E. (2016). Edible Insects and Young Adults in a North-East Italian City an Exploratory Study. Br. Food J..

[72] Nyberg M., Olsson V., Wendin K. (2020). Reasons for Eating Insects? Responses and Reflections among Swedish Consumers. Int. J. Gastron. Food Sci..

[73] Sogari G. (2015). Entomophagy and Italian Consumers: An Exploratory Analysis. Prog. Nutr..

[74] Berger S., Bärtsch C., Schmidt C., Christandl F., Wyss A.M. (2018). When Utilitarian Claims Backfire: Advertising Content and the Uptake of Insects as Food. Front. Nutr..

[75] Cavallo C., Materia V.C. (2018). Insects or Not Insects? Dilemmas or Attraction for Young Generations: A Case in Italy. Int. J. Food Syst. Dyn..

[76] Spartano S., Grasso S. (2021). UK Consumers’ Willingness to Try and Pay for Eggs from Insect-Fed Hens. Future Foods.

[77] Simeone M., Scarpato D. (2022). Consumer Perception and Attitude toward Insects for a Sustainable Diet. Insects.

[78] Verneau F., La Barbera F., Kolle S., Amato M., Del Giudice T., Grunert K. (2016). The Effect of Communication and Implicit Associations on Consuming Insects: An Experiment in Denmark and Italy. Appetite.

[79] Arena E., Mazzaglia A., Selvaggi R., Pecorino B., Fallico B., Serranò M., Pappalardo G. (2020). Exploring Consumer’s Propensity to Consume Insect-Based Foods. Empirical Evidence from a Study in Southern Italy. Appl. Syst. Innov..

[80] Kostecka J., Konieczna K., Cunha L.M. (2017). Evaluation of Insect-Based Food Acceptance by Representatives of Polish Consumers in the Context of Natural Resources Processing Retardation. J. Ecol. Eng..

[81] Menozzi D., Sogari G., Mora C., Gariglio M., Gasco L., Schiavone A. (2021). Insects as Feed for Farmed Poultry: Are Italian Consumers Ready to Embrace This Innovation?. Insects.

[82] Sogari G., Menozzi D., Mora C., Gariglio M., Gasco L., Schiavone A. (2022). How Information Affects Consumers’ Purchase Intention and Willingness to Pay for Poultry Farmed with Insect-Based Meal and Live Insects. J. Insects Food Feed..

[83] Barsics F., Caparros Megido R., Brostaux Y., Barsics C., Blecker C., Haubruge E., Francis F. (2017). Could New Information Influence Attitudes to Foods Supplemented with Edible Insects?. Br. Food J..

[84] Mancini S., Sogari G., Menozzi D., Nuvoloni R., Torracca B., Moruzzo R., Paci G. (2019). Factors Predicting the Intention of Eating an Insect-Based Product. Foods.

[85] Rumpold B.A., Langen N. (2019). Potential of Enhancing Consumer Acceptance of Edible Insects via Information. J. Insects Food Feed..

[86] Schouteten J.J., De Steur H., De Pelsmaeker S., Lagast S., Juvinal J.G., De Bourdeaudhuij I., Verbeke W., Gellynck X. (2016). Emotional and Sensory Profiling of Insect-, Plant- and Meat-Based Burgers under Blind, Expected and Informed Conditions. Food Qual. Prefer..

[87] Fasanelli R., Galli I., Riverso R., Piscitelli A. (2020). Social Representations of Insects as Food: An Explorative-Comparative Study among Millennials and X-Generation Consumers. Insects.

[88] Iannuzzi E., Sisto R., Nigro C. (2019). The Willingness to Consume Insect-Based Food: An Empirical Research on Italian Consumers. Agric. Econ..

[89] Berger S., Wyss A.M. (2020). Consumers’ Willingness to Consume Insect-Based Protein Depends on Descriptive Social Norms. Front. Sustain. Food Syst..

[90] Koch J.A., Bolderdijk J.W., van Ittersum K. (2021). Disgusting? No, Just Deviating from Internalized Norms. Understanding Consumer Skepticism toward Sustainable Food Alternatives. J. Environ. Psychol..

[91] Russell P.S., Knott G. (2021). Encouraging Sustainable Insect-Based Diets: The Role of Disgust, Social Influence, and Moral Concern in Insect Consumption. Food Qual. Prefer..

[92] Detilleux L., Wittock G., Dogot T., Francis F., Caparros Megido R. (2020). Edible Insects, What about the Perceptions of Belgian Youngsters?. Br. Food J..

[93] Iseppi L., Rizzo M., Gori E., Nassivera F., Bassi I., Scuderi A. (2021). Rasch Model for Assessing Propensity to Entomophagy. Sustainability.

[94] Sogari G., Menozzi D., Mora C. (2018). Sensory-Liking Expectations and Perceptions of Processed and Unprocessed Insect Products. Int. J. Food Syst. Dyn..

[95] Megido R.C., Gierts C., Blecker C., Brostaux Y., Haubruge É., Alabi T., Francis F. (2016). Consumer Acceptance of Insect-Based Alternative Meat Products in Western Countries. Food Qual. Prefer..

[96] Cicatiello C., Vitali A., Lacetera N. (2020). How Does It Taste? Appreciation of Insect-Based Snacks and Its Determinants. Int. J. Gastron. Food Sci..

[97] Ribeiro J.C., Santos C., Lima R.C., Pintado M.E., Cunha L.M. (2022). Impact of Defatting and Drying Methods on the Overall Liking and Sensory Profile of a Cereal Bar Incorporating Edible Insect Species. Future Foods.

[98] Smarzyński K., Sarbak P., Musiał S., Jezowski P., Piatek M., Kowalczewski P.T. (2019). Nutritional Analysis and Evaluation of the Consumer Acceptance of Pork Pâté Enriched with Cricket Powder-Preliminary Study. Open Agric..

[99] Tan H.S.G., Verbaan Y.T., Stieger M. (2017). How Will Better Products Improve the Sensory- Liking and Willingness to Buy Insect-Based Foods?. Food Res. Int..

[100] Tzompa-Sosa D.A., Dewettinck K., Gellynck X., Schouteten J.J. (2022). Consumer Acceptance towards Potato Chips Fried in Yellow Mealworm Oil. Food Qual. Prefer..

[101] Collins C.M., Vaskou P., Kountouris Y. (2019). Insect Food Products in the Western World: Assessing the Potential of a New “Green” Market. Ann. Entomol. Soc. Am..

[102] Halonen V., Uusitalo V., Levänen J., Sillman J., Leppäkoski L., Claudelin A. (2022). Recognizing Potential Pathways to Increasing the Consumption of Edible Insects from the Perspective of Consumer Acceptance: Case Study from Finland. Sustainability.

[103] Martins O.M.D., Bucea-Manea-Țoniș R., Coelho A.S., Simion V.-E. (2022). Sensory Perception Nudge: Insect-Based Food Consumer Behavior. Sustainability.

[104] Mandolesi S., Naspetti S., Zanoli R. (2022). Exploring Edible Insects’ Acceptance through Subjective Perceptions: A Visual Q Study. J. Insects Food Feed..

[105] Tan H.S.G., van den Berg E., Stieger M. (2016). The Influence of Product Preparation, Familiarity and Individual Traits on the Consumer Acceptance of Insects as Food. Food Qual. Prefer..

[106] Bruckdorfer R.E., Büttner O.B. (2022). When creepy crawlies are cute as bugs: Investigating the effects of (cute) packaging design in the context of edible insects. Food Qual. Prefer..

[107] Reverberi M. (2021). The New Packaged Food Products Containing Insects as an Ingredient. J. Insects Food Feed..

[108] Gallen C., Pantin-Sohier G., Oliveira D. (2022). How Can the Design Thinking Process Improve an Innovative Insect-Based Food Experience?. Int. J. Food Des..

[109] Herbert M., Beacom E. (2021). Exploring Consumer Acceptance of Insect-based Snack Products in Ireland. J. Food Prod. Mark..

[110] Berger S., Christandl F., Schmidt C., Baertsch C. (2018). Price-Based Quality Inferences for Insects as Food. Br. Food J..

[111] Powell P.A., Jones C.R., Consedine N.S. (2019). It’s Not Queasy Being Green: The Role of Disgust in Willingness-to-Pay for More Sustainable Product Alternatives. Food Qual. Prefer..

[112] Lippi N., Predieri S., Chieco C., Daniele G.M., Cianciabella M., Magli M., Maistrello L., Gatti E. (2021). Italian Consumers’ Readiness to Adopt Eggs from Insect-Fed Hens. Animals.

[113] Spartano S., Grasso S. (2021). Consumers’ Perspectives on Eggs from Insect-Fed Hens: A UK Focus Group Study. Foods.

[114] Ankamah-Yeboah I., Jacobsen J.B., Olsen S.B. (2018). Innovating out of the Fishmeal Trap: The Role of Insect-Based Fish Feed in Consumers’ Preferences for Fish Attributes. Br. Food J..

[115] Popoff M., MacLeod M., Leschen W. (2017). Attitudes towards the Use of Insect-Derived Materials in Scottish Salmon Feeds. J. Insects Food Feed..

[116] Onwezen M.C., van den Puttelaar J., Verain M.C.D., Veldkamp T. (2019). Consumer Acceptance of Insects as Food and Feed: The Relevance of Affective Factors. Food Qual. Prefer..

[117] Lunden S., Hopia A., Forsman L., Sandell M. (2020). Sensory and Conceptual Aspects of Ingredients of Sustainable Sources-Finnish Consumers’ Opinion. Foods.

[118] Fischer A.R.H. (2021). Eating Insects—From Acceptable to Desirable Consumer Products. J. Insects Food Feed..

[119] Lorini C., Ricotta L., Vettori V., Del Riccio M., Biamonte M.A., Bonaccorsi G. (2021). Insights into the Predictors of Attitude toward Entomophagy: The Potential Role of Health Literacy: A Cross-Sectional Study Conducted in a Sample of Students of the University of Florence. Int. J. Environ. Res. Public Health.

[120] Berger S., Christandl F., Bitterlin D., Wyss A.M. (2019). The Social Insectivore: Peer and Expert Influence Affect Consumer Evaluations of Insects as Food. Appetite.

[121] Milani Marin L.E., Jacomuzzi A.C. (2020). Insects at the Table: What Consumers Know. Riv. Di Studi Sulla Sostenibilita.

[122] Marin L.E.M., Sanjinez J.O.S.P., Jacomuzzi A.C. (2021). Insects as Food: Knowledge, Desire and Media Credibility. Ideas for a Communication. Riv. Di Studi Sulla Sostenibilita.

[123] Hartmann C., Ruby M.B., Schmidt P., Siegrist M. (2018). Brave, Health-Conscious, and Environmentally Friendly: Positive Impressions of Insect Food Product Consumers. Food Qual. Prefer..

[124] Sidali K.L., Pizzo S., Garrido-Pérez E.I., Schamel G. (2019). Between Food Delicacies and Food Taboos: A Structural Equation Model to Assess Western Students’ Acceptance of Amazonian Insect Food. Food Res. Int..

[125] Poortvliet P.M., van der Pas L., Mulder B.C., Fogliano V. (2019). Healthy, but Disgusting: An Investigation into Consumers’ Willingness to Try Insect Meat. J. Econ. Entomol..

[126] Stone H., FitzGibbon L., Millan E., Murayama K. (2022). Curious to Eat Insects? Curiosity as a Key Predictor of Willingness to Try Novel Food. Appetite.

